# Analysis of Three-Dimensional Tooth Movement: A Comparative Study Between Digital Dental Models and Craniofacial Models

**DOI:** 10.7759/cureus.67094

**Published:** 2024-08-17

**Authors:** Rie Kubota, Kenji Fushima, Hirofumi Arisaka

**Affiliations:** 1 Department of Anesthesiology, Graduate School of Dentistry, Kanagawa Dental University, Yokosuka, JPN; 2 Dentistry and Orthodontics, Kanagawa Dental University, Yokosuka, JPN

**Keywords:** orthognathic surgery, tooth movement analysis, cranial region of interest, palatal region of interest, craniofacial model, digital dental model

## Abstract

Objective: This study aims to validate the efficacy of using a digital dental model (DM) with reference to the palatal region of interest (PROI) for assessing orthodontic tooth movement (TM) by comparing it with the analysis of a computed tomography (CT) model with reference to the cranial region of interest (CROI).

Materials and methods: Thirty-four patients (mean age: 21 years and 11 months) with jaw deformities underwent DM and CT scans before and after presurgical orthognathic treatment. Linear and angular measurements during TM were conducted in three dimensions using both DM and CT to assess reliability.

Results: DM analysis with PROI registration exhibited high levels of reproducibility, with minimal standard errors in X, Y, and Z displacements (<0.15 mm) and 0.43 degrees in angular change. CT analysis with CROI registration demonstrates similarly high reproducibility, with standard errors inferior to DM analysis (<0.20 mm). Bland-Altman analysis indicated agreement in linear changes of each X, Y, and Z displacement between DM and CT measurements, with limits of agreement (LOA) below 0.91 mm.

Conclusions: The results of this study suggest that the PROI, focusing on the third palatal rugae and the horizontal part of the palatal vault, serves as a reliable reference region for evaluating three-dimensional (3D) tooth movement.

Clinical significance: Digital dental models offer distinct advantages including the absence of X-ray exposure, no metal artifacts, and the ability to generate high-resolution 3D models. The methodology demonstrated high precision and reproducibility, supporting its potential clinical utility in orthodontic treatment planning and assessment.

## Introduction

In orthodontic care, it is crucial to conduct both long-term and short-term assessments of tooth movement (TM) to ensure the effectiveness of the treatment plan and applied mechanics. Traditionally, TM has been assessed through the two-dimensional (2D) analysis of lateral cephalograms [1]. To evaluate TM by lateral cephalograms, the tracing superimposition before and after treatment is usually performed using cranial reference planes such as the Sella-Nasion (SN) plane, Frankfort horizontal (FH) plane, and basion-nasion (Ba-Na) plane. In recent years, the clinical application of three-dimensional (3D) medical images such as optical impression and cone beam computed tomography (CBCT) has become widespread in the orthodontic field [2,3].

To assess 3D TM in orthodontics, Cevidanes et al. [4] reported on cranial registration using craniofacial models reconstructed from CBCT in growing patients. The study concluded that superimposition with respect to the orbit, infraorbital foramen, and zygomatic bone is a viable method for analyzing TM before and after treatment. Nada et al. [5] reported that voxel-based image registration on the anterior cranial base and zygomatic arch could be considered an accurate and reproducible method for CBCT superimposition. With the expanding clinical application of 3D medical imaging in dentistry, cranial registration using CT or CBCT has become an important method to evaluate TM and jaw displacement [3-7].

The use of a digital dental model (DM) for diagnosis and treatment evaluation is being considered [3,8,9]. DM is devoid of risks associated with X-ray exposure and can be readily obtained in daily clinical practice, indicating its potential for future clinical applications. By utilizing DM, TM is capable of conducting 3D evaluations, and the effectiveness of superimposing DM before and after treatment based on the palatal configuration has been reported [9-17]. Bailey et al. [10] investigated the impact of orthodontic treatment on palatal rugae morphology. They concluded that the medial and lateral points of the third palatal rugae serve as stable landmarks for anatomical reference in longitudinal cast analysis. It was reported that the reliable area of superposition of serial dental models was evaluated with reference to miniscrews. Jang et al. [14] highlighted the reliability of using the medial points of the third palatal rugae and the palatal vault as a reference region for assessing tooth movement. Chen et al. [15] identified the medial two-thirds of the third rugae and the palatal vault dorsal to it as a stable region for assessing orthodontic tooth movement in adult patients. Their study is deemed reliable as it refers to unloaded miniscrews to identify stable regions.

A comparative study with 2D cephalometric analysis was reported to assess the reproducibility and accuracy of 3D TM analysis using DM. Cha et al. [16] compared the tooth movement results obtained by 3D surface-to-surface matching of the palate with those of cephalometric analysis and reported a high degree of agreement between the two, but the palatal reference region is considered unclear. Liu et al. [17] reported that 3D digital superimpositions of dental models were clinically as reliable as cephalometric superimpositions in assessing tooth movements. These studies were limited to 2D assessment. To validate TM analysis in 3D, the plan is to compare 3D DM analysis with the results obtained from another measurement equipment's 3D analysis.

In our daily clinical practice, we utilize DM, with a specific focus on the palatal region of interest (PROI), to assess TM. To enhance the applicability of DM in examining TM within orthodontic clinical practice, it seems essential to evaluate the validity of DM analysis with PROI registration in 3D. This study aimed to further validate the utility of PROI as a reference region for assessing TM. The investigation involved the use of 3D CT models and DM obtained at the initial and presurgical recording from orthognathic surgery cases.

We aim to examine the precision and measurement error of 3D TM analysis with PROI registration of DM before and after the preoperative treatment period, examine the precision and measurement error of 3D TM analysis with reference to the cranial region of interest (CROI) set by CT before and after the preoperative treatment period, and compare the 3D TM between DM analysis with PROI registration and CT analysis with CROI registration.

## Materials and methods

Patients

The subjects of this study were selected from consecutive patients who visited the Department of Orthodontics from 2013 to 2021. They were diagnosed with jaw deformity and underwent orthodontic treatment with orthognathic surgery.

Patients who had been treated by means of the multi-bracket appliance without extraction of any teeth in the maxillary dentition except for the third molar were included. Patients who completed preoperative orthodontic treatment were selected, and the following exclusion criteria were used for case selection: patients with orthodontic implants on the palatal side, patients who underwent skeletal maxillary expansion, patients with cleft lip and palate-associated malocclusions, and patients with high metal artifacts in the CT images.

The present study ultimately enrolled 34 patients (13 men and 21 women) with a mean age at the time of their initial examination of 21 years and 11 months, ranging from 15 to 38 years. In patients under 20 years of age, systemic evaluation showed no changes in height, and lateral cephalometric analysis indicated no mandibular growth during the preoperative orthodontic period. Consequently, growing patients were excluded from the study. The demographic characteristics of the patients are shown in Table 1. Of these 34 cases, according to Angle's classification, four patients were seen to exhibit class II malocclusion, 18 class III malocclusion, one class I and II malocclusion, and 11 class I and III malocclusion.

**Table 1:** Study participant protocols

Study participants		Number (%)
Total visited patients	2021-2014	1,621 (100)
Orthognathic surgery cases		571 (35.2)
Exclusion criteria	Orthodontic implant, maxillary expansion, metal artifact	34 (2.10)
Sex (male/female)		13 (0.80)/21 (1.30)
Angle's classification	Class Ⅱ	4 (0.25)
	Class Ⅲ	18 (1.11)
	Asymmetrical class Ⅱ	1 (0.06)
	Asymmetrical class Ⅲ	11 (0.68)
	Total	34 (2.10)

Ethical approval for the use of the data and to conduct the research was obtained (date of approval: August 12, 2022).

Methods

Plaster dental casts and computed tomography (CT) at the time of initial examination (T0) and at the time of examination before orthognathic surgery (T1) (after preoperative orthodontic treatment) were taken for diagnostic purposes and used in this research. The average duration of preoperative orthodontic treatment was 16.3 months, ranging from seven months to 45 months.

Three-Dimensional Models

The plaster cast models of the maxillary dentition were measured in three dimensions using a 3D scanner C-Pro Dental System D800-3SP (Panasonic Corporation, Osaka, Japan) and saved as STL-format data. DICOM-format data of the craniofacial region were acquired from the CT scanner Medical Alexion Advance TSX-034A/1B (Toshiba, Tochigi, Japan). The CT imaging employed a 512 × 512 matrix with a pixel size of 0.4687 mm × 0.4687 mm, a field of view (FOV) of 240 mm, and a slice thickness of 3.0 mm. 3D diagnostic software originally developed using the programming language Visual C++ was employed to reconstruct 3D digital models of the maxillary dentition and craniofacial dentoskeletal models [3]. The 3D dental digital models at the initial visit and before orthognathic surgery were defined as DM-T0 and DM-T1, respectively. Similarly, the 3D craniofacial models at the first visit and before orthognathic surgery were defined as CT-T0 and CT-T1, respectively.

Palatal Region of Interest

**Figure 1:**
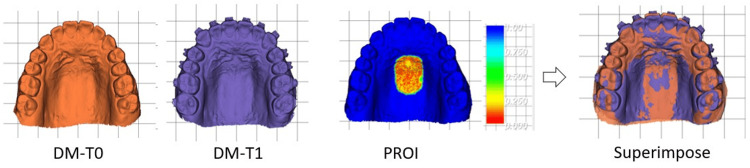
PROI registration The PROI, which encompassed the area surrounding the third palatal ruga and the horizontal portion of the palatine vault, was used to superimpose DM-T0 and DM-T1. The color map area indicates the PROI point-cloud distance between DM-T0 and DM-T1. An RGB color scale bar (in millimeters) corresponding to the point-cloud distance is shown on the right. PROI: palatal region of interest, DM-T0: digital dental model constructed at the initial visit, DM-T1: digital dental model constructed before orthognathic surgery, RGB: red/green/blue

DMs were employed to analyze 3D TM during preoperative orthodontic treatment. As shown in Figure 1, the DM-T1 image was superimposed on the DM-T0 image with reference to the palatal region of interest (PROI) defined as the hard palate that encompasses and extends posteriorly from the third palatal rugae. Posteriorly, the PROI extends close to the distal-proximal aspect of the first molar but does not reach the junction of the hard and soft palates. Laterally, the PROI is the horizontal region of the hard palate approximately less than 10 mm away from the median palatal raphe.

Superimposition was performed using the iterative closest point (ICP) algorithm, which is a local superimposition without feature points [18,19]. The color map area indicates the PROI point cloud distance between DM-T0 and DM-T1. Visually, PROI overlap was good with a point cloud distance of less than 0.5 mm in this example.

Cranial Region of Interest

**Figure 2:**
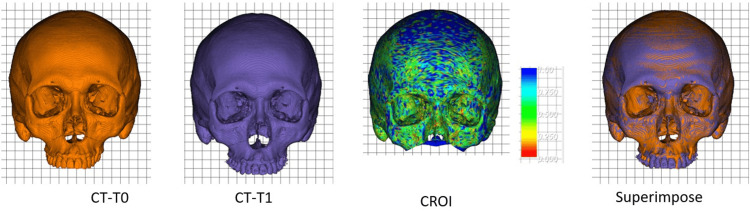
CROI registration The CROI was defined as the entire cranium, including the orbit. CT-T1 was superimposed on CT-T0 with reference to the CROI. CROI: cranial region of interest, CT-T0: 3DCT model at the initial visit, CT-T1: 3DCT model before orthognathic surgery, 3DCT: three-dimensional computed tomographic

3D TM during preoperative orthodontic treatment was also analyzed using CT models with cranial registration using the ICP algorithm. As shown in Figure 2, CT-T1 was superimposed on the CT-T0 with reference to the cranial region of interest (CROI) defined as the entire cranium including the orbit. In this example, the majority of the color map in CROI registration is shown in green at 0.5 mm, indicating a good fit.

Three-Dimensional Analysis of Tooth Movement

3D TM of the maxillary dentition was analyzed using DM with PROI registration and CT model with CROI registration. The teeth analyzed were the maxillary right central incisor and left first molar. Metal artifacts were observed on the CT-T1 due to brace wear at T1 recording. In cases where metal artifacts were present unilaterally in the CT model, the contralateral side with better image quality was selected. However, if metal artifacts were present bilaterally in the CT model, the tooth was excluded from the study. Ultimately, the numbers of central incisors and first molars were 34 and 25, respectively.

**Figure 3:**
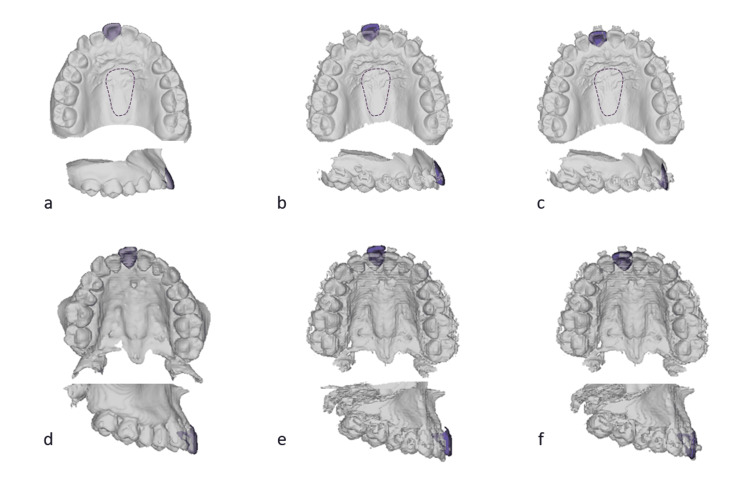
Three-by-three transformation matrix for tooth movement analysis After superimposing DM-T1 onto DM-T0 using PROI registration (a-c) and CT-T1 onto CT-T0 using CROI registration (d-f), the crown surface of each tooth (a and d) was extracted from the T0 model (b and e) and aligned with the corresponding crown of the T1 model using the ICP method (c and f). A three-by-three transformation matrix was derived for each target tooth. PROI: palatal region of interest, CROI: cranial region of interest, DM-T0: digital dental modeling at the initial visit, DM-T1: digital dental model before orthognathic surgery; CT-T0: 3DCT model at the initial visit, CT-T1: 3DCT model before orthognathic surgery, 3DCT: three-dimensional computed tomographic

In the DM analysis, following the superimposition of DM-T1 onto DM-T0 through PROI registration, the crown of the target tooth was extracted from the DM-T0, excluding the cervical and adjacent surfaces (Figure 3a). The crown of DM-T0 was superimposed with the corresponding tooth of DM-T1 using the ICP method, and consequently, a three-by-three transformation matrix was derived for each target tooth (Figure 3b, 3c). In the 3DCT model, a similar process was performed following the superimposition of CT-T1 onto CT-T0 through CROI registration (Figure 3d-3f). The transformation matrix, derived by aligning the tooth crown from the T0 model onto the T1 model, was employed for the subsequent 3D TM analysis.

Occlusal Plane Coordinate System

**Figure 4:**
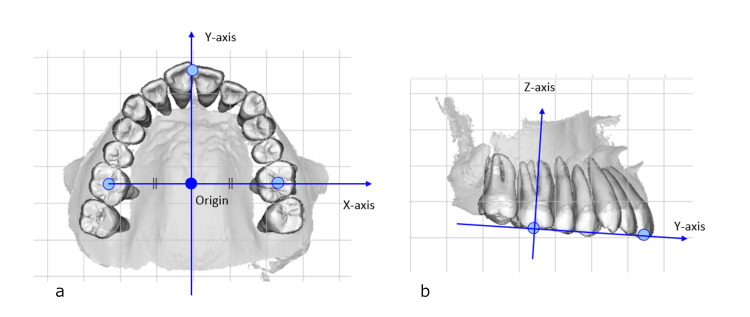
Occlusal plane coordinate system Origin: midpoint of the left/right M1 passive centric (M1-PC), X-axis: line connecting the left/right M1-PC, XY plane: plane including the X-axis and the midpoint of the left/right mesial point of the central incisor, Y-axis: line through the origin and perpendicular to the X-axis, Z-axis: line through the origin and perpendicular to the XY plane a: occlusal view, b: sagittal view

Figure 4 describes the occlusal plane coordinate system for analyzing TM. On the DM-T0 or CT-T0, the coordinates of the mesial point of the central incisor (M-I1) and the passive centric of the first molar (M1-PC) were measured bilaterally. The origin of the coordinate system was defined as the midpoint coordinates of the right and left M1-PC. A straight line passing through the right and left M1-PC was defined as the X-axis, and the plane containing the X-axis and passing through the midpoint of the right and left M-I1 was defined as the XY plane. A line through the origin and perpendicular to the X-axis in the XY plane was defined as the Y-axis. A straight line passing through the origin and perpendicular to the XY plane was defined as the Z-axis.

Linear and Angle Measurements

**Figure 5:**
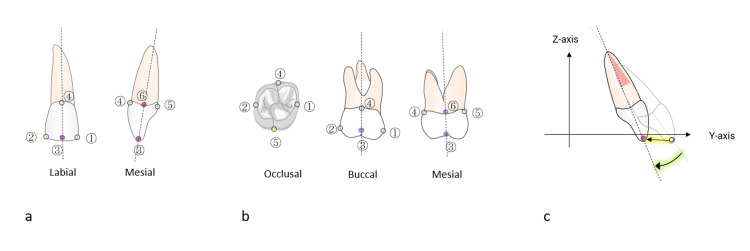
Linear and angle measurements After the registration between the T0 and T1 models with reference to the PROI or CROI, linear and angle measurements were made. a: landmark points on the right central incisors; b: landmark points on the left first molar; ① mesial point, ② distal point, and ③ midpoint between the mesial and distal points; ④ labial/buccal point, ⑤ lingual point, and ⑥ midpoint between the labial/buccal and lingual points; dotted line: virtual tooth axis, which was defined as a straight line connecting points ③ and ⑥; c: three-dimensional movement of the crown point from T0 to T1 analyzed for each X, Y, and Z coordinate The changes in angle in the virtual tooth axis were evaluated in the YZ plane. PROI: palatal region of interest, CROI: cranial region of interest

Figure 5a and Figure 5b represent landmark points and tooth axes of the target teeth measured on the T0 model. The coordinates of the mesial and distal points were measured at the incisal edge of the central incisors, the marginal ridges of the first molars, while the coordinates of the labial/buccal and lingual cervical gingival points were measured. The midpoint between the mesial and distal points was obtained as the coronal point, and the midpoint between the labial/buccal and lingual points was obtained as the cervical point. A straight line connecting the coronal point and the cervical point was set as the virtual tooth axis.

Following the registration between T0 and T1 models with reference to PROI or CROI, linear and angular measurements were performed. The measurement points initially established on the T0 model were replicated onto the T1 model using transformation matrices obtained by aligning the tooth crown from the T0 model onto the T1 model. As a result, the virtual tooth axis was faithfully reproduced on the T1 model (Figure 5c) [20]. 3D movements of the coronal points from T0 to T1 were analyzed for each X, Y, and Z coordinate. The angular change of the virtual tooth axis was examined in the YZ plane.

Reliability Tests

The measurement error in this study, including all processes using three-dimensional analysis software, superimposition of the T0 model and T1 model, measurement on a PC screen, and tooth movement superimposition, was determined by the following method.

Linear and angular measurements were performed using both DM with PROI registration and CT with CROI registration for each target tooth in 34 subjects. One week after the first measurement, the same measurements were repeated by the same researcher (RK) to eliminate inter-rater error. Reliability tests were conducted for the following cases: the reproducibility and measurement error between the first and second measurements in DM analysis with reference to PROI, the reproducibility and measurement error between the first and second measurements in CT analysis with reference to CROI, and the comparison between DM analysis employing PROI registration and CT analysis utilizing CROI registration.

The measurement error between the first and second measurements and between the DM analysis and CT analysis was calculated using the following formula: standard error (SE) = √(Σd2 / 2n), where d represents the difference between the two measurements and n represents the number of cases. The reliability coefficient was calculated using the Houston formula [21].

A simple linear regression analysis was performed to test the correlation between the first and second measurements and between PROI and CROI registrations. Bland-Altman analysis was performed to compare the differences in measurements between PROI and CROI registrations [22]. All statistical analyses were performed using a software package (SPSS version 21.0, IBM SPSS Statistics, Armonk, NY). Statistical significance was set at p < 0.01.

## Results

Reproducibility of and measurement error associated with DM with reference to the PROI

**Figure 6:**
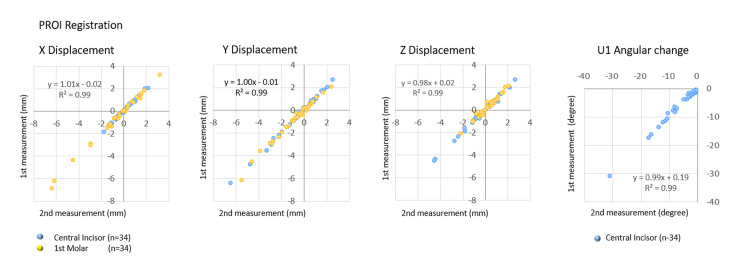
Reproducibility of three-dimensional tooth movements, assessed by DM analysis with reference to the PROI Scatterplot illustrating the relationships of the first and second measurements of the linear displacements of the X, Y, and Z axes with the change in the angle of each tooth. DM: digital dental model, PROI: palatal region of interest, U1: maxillary central incisor

The reproducibility of linear changes in the crown points of each target tooth and angular change of the central incisor was analyzed using DM with PROI registration. Scatterplots between the first and second measurements, as depicted in Figure 6, indicated strong correlations in each X, Y, and Z displacement and angular change, with correlation determination (R2) consistently at 0.99.

As indicated in Table 2, there were no significant differences in the linear measurement changes in X, Y, and Z displacements, as well as the angular change of the central incisor, between the first and second measurements. The standard error in X, Y, and Z displacements of the central incisor and the first molar was less than 0.09 mm and 0.15 mm, respectively, with reliability coefficients exceeding 97.8%. The standard error in the angular change of the central incisor was 0.43 degrees, with a reliability coefficient of 99.6%.

**Table 2:** Reproducibility of and measurement error associated with DM with reference to PROI DM: digital dental model, PROI: palatal region of interest, U1: maxillary central incisor, M1: maxillary first molar, SD: standard deviation, Diff.: difference between the first and second measurement, NS: no significant difference at the 0.01% level

		Number	1st mean±SD	2nd mean±SD	Diff.	T	SE	Reliability coefficient (%)
U1	X displacement	34	0.17±0.88	0.16±0.88	0.01±0.08	-0.40 NS	0.06	99.5
	Y displacement	34	-0.32±1.97	-0.29±1.99	-0.03±0.11	-0.07 NS	0.08	99.9
	Z displacement	34	-0.21±1.82	-0.23±1.80	-0.004±0.16	1.56 NS	0.09	99.8
	Angular change (degree)	34	-5.89±6.57	-5.63±6.52	-0.25±0.61	-2.42 NS	0.43	99.6
M1	X displacement	34	-0.59±2.06	-0.63±2.10	0.03±0.15	1.27 NS	0.11	99.7
	Y displacement	34	-0.68±1.91	-0.72±1.89	0.04±0.20	0.62 NS	0.15	99.4
	Z displacement	34	0.19±0.94	0.24±0.91	-0.05±0.20	0.44 NS	0.14	97.8
U1	Angular change (degree)	34	-5.89±6.57	-5.89±6.57	-0.25±0.61	-2.42 NS	0.43	99.6

Reproducibility and measurement in CT analysis with reference to CROI

**Figure 7:**
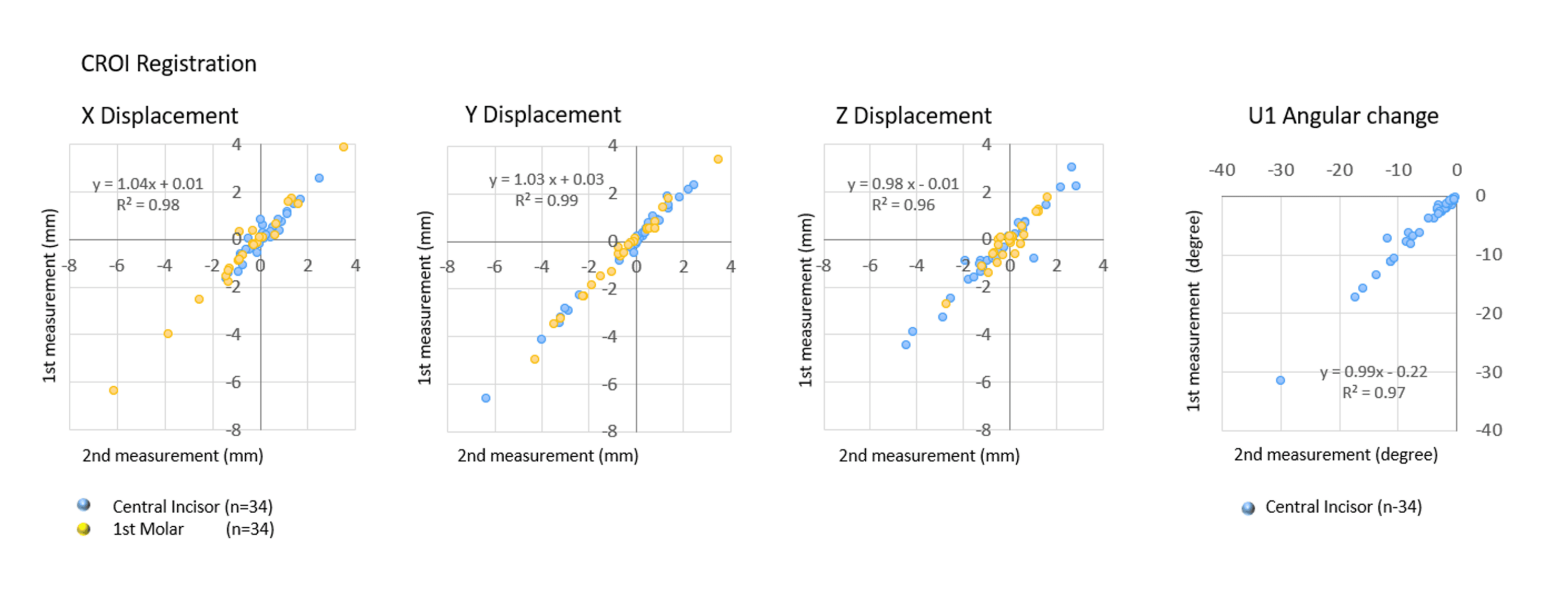
Reproducibility of the three-dimensional tooth movement data obtained using CT with reference to CROI Scatterplots illustrating the relationships between the first and second measurements of the linear displacements on the X, Y, and Z axes and the change in angle. CT: computed tomography, CROI: cranial region of interest, U1: maxillary central incisor

Similarly, the reproducibility of linear changes in the crown points of each target tooth and angular change of the central incisor was analyzed using CT with CROI registration. As shown in Figure 7, scatterplots of the linear and angular changes between the first and second measurements revealed good correlations in each X, Y, and Z displacement and angular change, with R2 values exceeding 0.96.

As indicated in Table 3, there were no significant differences in the linear measurement changes in X, Y, and Z displacements, as well as the angular change of the central incisor, between the first and second measurements. The standard error in X, Y, and Z displacements of the central incisor and the first molar was less than 0.19 mm and 0.20 mm, respectively, with reliability coefficients exceeding 93.9%. The standard error in the angular change of the central incisor was 0.75 degrees, with a reliability coefficient of 99.1%.

**Table 3:** Reproducibility of and measurement errors in CT analysis performed with reference to the CROI CT: computed tomography, CROI: cranial region of interest, U1: maxillary central incisor, M1: maxillary first molar, SD: standard deviation, Diff.: difference between the first and second measurement, NS: no significant difference at the 0.01% level

		Number	1st mean±SD	2nd mean±SD	Diff.	T	SE	Reliability coefficient (%)
U1	X displacement	34	0.19±0.87	0.16±0.92	0.02±0.24	0.47 NS	0.17	96.2
	Y displacement	34	-0.30±1.93	-0.29±1.99	-0.02±0.17	-0.54 NS	0.12	99.6
	Z displacement	34	-035±1.85	-0.28±1.85	0.07±0.27	-1.47 NS	0.19	98.9
	Angular change (degree)	34	-5.47±6.07	-5.61±6.07	0.15±0.75	0.80 NS	0.75	99.1
M1	X displacement	25	0.25±1.69	0.22±22.91	0.02±0.11	-0.61 NS	0.14	99.2
	Y displacement	25	-0.47±1.95	-0.47±1.96	-0.01±0.16	-0.50 NS	0.14	99.0
	Z displacement	25	0.04±1.43	0.04±1.43	-0.004±0.16	0.98 NS	0.20	93.9
U1	Angular change (degree)	34	-5.47±6.07	-5.61±6.07	-0.15±0.75	0.80 NS	0.75	99.1

Concordance between the results of DM and CT analysis

**Figure 8:**
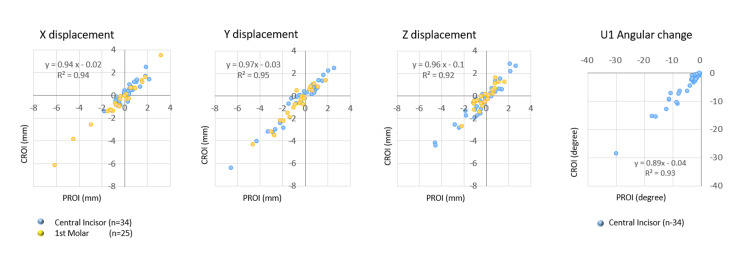
Comparison of the three-dimensional tooth movements identified using DM and CT analysis Scatterplot illustrating the relationship between data obtained using DM with PROI registration and CT analysis with CROI registration for the linear displacements along the X, Y, and Z axes and the change in angle. DM: digital dental model, CT: computed tomography, PROI: palatal region of interest, CROI: cranial region of interest, U1: maxillary central incisor

3D tooth movement analysis using DM with PROI registration was compared to CT analysis with CROI registration. As shown in Figure 8, scatterplots illustrating the linear changes in each X, Y, and Z displacement, along with the angular change during preoperative orthodontics between DM analysis and CT analysis, exhibited strong correlations, with R2 values exceeding 0.92.

As indicated in Table 4, there were no significant differences in the linear measurement changes in X, Y, and Z displacements, as well as the angular change of the central incisor, between DM analysis and CT analysis. Based on the CT analysis, the standard error of the DM analysis in X, Y, and Z displacements of the central incisor and the first molar was less than 0.33 mm and 0.24 mm, respectively, with reliability coefficients below 88.9%. The standard error in the angular change of the central incisor was 1.79 degrees, with a reliability coefficient of 96.2%.

**Table 4:** Concordance between the data obtained using DM (PROI registration) and CT analysis (CROI registration) DM: digital dental model, PROI: palatal region of interest, CT: computed tomography, CROI: cranial region of interest, U1: maxillary central incisor, M1: maxillary first molar, SD: standard deviation, Diff.: difference between the first and second measurement, NS: no significant difference at the 0.01% level

		Number	PROI mean±SD	CROI mean±SD	Diff.	T	SE	Reliability coefficient (%)
U1	X displacement	34	0.17±0.88	0.20±0.87	-0.03±0.37	-0.40 NS	0.26	91.1
	Y displacement	34	-0.31±1.93	-0.30±1.93	-0.005±0.41	0.06 NS	0.28	97.8
	Z displacement	34	-0.21±1.81	-0.33±1.85	0.12±0.46	-0.07 NS	0.33	96.7
	Angular change (degree)	34	-5.89±6.56	-5.32±6.02	-0.56±1.82	-1.95 NS	1.79	96.2
M1	X displacement	25	-0.46±1.94	-0.54±1.85	0.08±0.31	1.27 NS	0.19	98.7
	Y displacement	25	-0.48±1.72	-0.43±1.71	0.05±0.40	0.62 NS	0.24	97.2
	Z displacement	25	-0.05±0.87	-0.09±0.94	0.04±0.41	0.44 NS	0.24	88.9
U1	Angular change (degree)	34	-5.89±6.56	-5.32±6.02	-0.56±1.82	-1.95 NS	1.79	96.2

**Figure 9:**
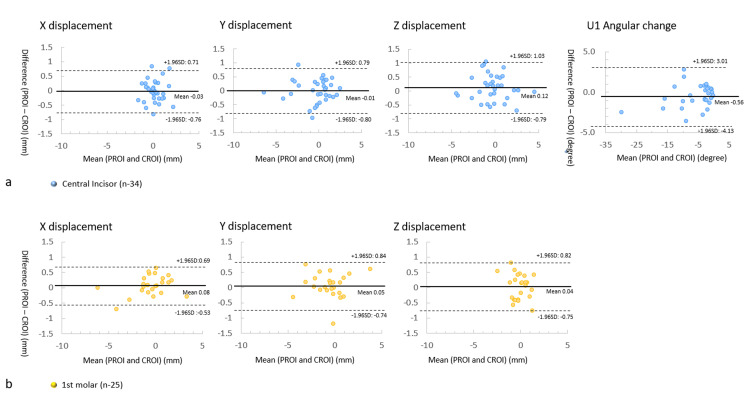
Comparison of the results of the DM and CT analysis Bland-Altman analysis was performed to compare the data obtained using DM with PROI registration and CT analysis with CROI registration. a: Differences in the linear displacements along the X, Y, and Z axes and the change in the angle of the central incisor. b: Differences in the linear displacements along the X, Y, and Z axes of the first molar. The solid lines represent the mean differences, and the dotted lines represent the limits of agreement. DM: digital dental model, PROI: palatal region of interest, CT: computed tomography, CROI: cranial region of interest, U1: maxillary central incisor

Figure 9 presents the Bland-Altman results. The vertical axis illustrates the difference value, calculated by subtracting the CT measurement value from the DM measurement value, while the horizontal axis represents the mean value of the DM and CT measurement values. Bland-Altman analysis revealed agreement in linear changes of each X, Y, and Z displacement between DM and CT measurements. For the central incisor (Figure 9a), the mean differences in linear changes for each X, Y, and Z displacement were -0.03 mm, -0.01 mm, and 0.12 mm with limits of agreement (LOA) of 0.73 mm, 0.80 mm, and 0.91 mm, respectively. The mean difference in the angular change was -0.56 degrees with LOA of 3.56 degrees. For the first molar (Figure 9b), the mean differences in linear changes for each X, Y, and Z displacement were 0.08 mm, 0.05 mm, and 0.04 mm, with LOA of 0.61 mm, 0.79 mm, and 0.78 mm, respectively.

## Discussion

In the analysis of 3D tooth movement using DM, establishing a reference region for the registration of serial dental models is crucial. Studies have suggested that considering palatal morphology is essential in this regard [10-17]. The systematic review conducted by Stucki and Gkantidis [13] on serial digital 3D dental model superimposition concluded that the medial two-thirds of the third fold along with the area 5 mm dorsal to them are among the reliable areas for palatal superimposition. In this study, PROI is defined as the horizontal region of the hard palate that encompasses and extends posteriorly from the third palatal rugae. The definition is considered reasonable in light of previous reports.

The aim of this study was to assess the validity of PROI as a registration region for serial dental models in evaluating tooth movement. The reproducibility and accuracy of tooth movement analysis using digital dental models were assessed through comparative studies with cephalometric analysis [16,17]. In contrast to these studies, the present study undertook a comparison with CT model analysis. This research holds significance as it facilitates the examination of three-dimensional data, and the accuracy of the cranial overlay referenced by CT is acknowledged to be high [4-7].

Reproducibility of 3D DM analysis with PROI registration

The precision of linear changes in crown points of target teeth and angular change of the central incisor, analyzed through PROI registration, exhibited high levels of reproducibility, with a correlation determination surpassing 0.99. The standard errors were minimal, indicating excellent reliability coefficients. These results suggest that the PROI registration method provides good reproducible measurements of three-dimensional tooth movement.

The reproducibility of the DM analysis in this study would primarily hinge on the PROI superimposition. This is because the superimposition of the target tooth crown by the ICP algorithm is assumed to possess high precision and accuracy.

Several studies have reported good reproducibility in the palatal superimposition of the serial dental models. Jang et al. [14] examined the displacement of the central incisor using the ruga-palate-superimposition method and confirmed its high reproducibility with an intra-class correlation coefficient of 0.998. Talaat et al. [12] reported acceptable repeatability, with intercorrelation coefficients exceeding 0.90 in the landmark-based palatal superimposition method. Chen et al. [15] investigated the displacement of the incisor and molar with reference to the medial two-thirds of the third rugae and the palatal vault dorsal to it. The study confirmed a high intra-class correlation coefficient greater than 0.99. Consistent with findings from other studies, the results of this research underscore the reproducibility of PROI registration in dental model analysis, effectively capturing three-dimensional tooth movement during preoperative orthodontic treatment.

Reproducibility of 3D CT analysis with CROI registration

Similarly, the CROI registration method exhibited high precision in linear changes and angular change, with a correlation determination exceeding 0.96. The standard errors were low, accompanied by high reliable coefficients.

Although the correlation coefficients and standard errors of linear and angular changes were satisfactory, the results were slightly inferior to those observed in the PROI analysis. The CROI registration appears to be reliable, attributed to the extensive registration area illustrated in Figure 2. On the contrary, the CT-reconstructed dentition image seems visually less accurate compared to the digital dental model, with the dentition image of CT-T1 particularly affected by brace wearing. The superposition of crowns in the CT images is deemed to be less accurate than that in the DM analysis (Figure 3), consequently impacting the overall reproducibility of the 3D tooth movement analysis with CROI registration.

Pan et al. [18] validated 3D maxillary DM superimposition and CBCT maxillary superimposition. Reportedly, the intra-examiner correlation coefficient for 3D tooth movement analysis in CBCT cranial registration surpasses 0.99, indicating a superiority over our obtained results. Notably, in our research, post-treatment recordings involved patients undergoing treatment (post-presurgical orthodontic treatment) while wearing braces, potentially contributing to the observed differences with the CBCT study.

Three-dimensional tooth movement analysis using CROI was deemed to be a reasonably effective method, albeit with slightly lower reproducibility compared to that of PROI.

Comparison of 3D TM analysis using DM (PROI registration) and CT (CROI registration)

The accuracy of 3D tooth movement analysis with PROI registration was assessed based on tooth movement with CROI registration. The linear and angular changes of the central incisors between PROI and CROI registrations revealed a high correlation determination surpassing 0.92. The lack of significant differences in linear and angular changes between the two registrations underscores the reliability of PROI as a reference region for three-dimensional tooth movement analysis.

The Bland-Altman analysis revealed consistent agreement in linear measurements between PROI and CROI registrations. The limits of agreement for each displacement along the X, Y, and Z axes were less than 0.91 mm and 0.79 mm, for the central incisor and first molar, respectively. Angular measurement also showed agreement between PROI and CROI registration with a limit of agreement of 3.56 degrees. These results further support the robustness of the methodology employed in this study.

In recent years, CBCT has gained prominence within the dental profession, offering a valuable tool for assessing tooth movement in orthodontic treatments. It has been reported that tooth movement analysis using CBCT has high reproducibility and accuracy and is superior to analysis using digital dental models [23]. Nevertheless, digital dental models offer distinct advantages including the absence of X-ray exposure, no metal artifacts, and the ability to generate high-resolution 3D models.

The results of this study suggest that the PROI, focusing on the third palatal rugae and the horizontal part of the palatal vault, serves as a reliable reference region for evaluating three-dimensional tooth movement. The methodology demonstrated high precision and reproducibility, supporting its potential clinical utility in orthodontic treatment planning and assessment.

The DM used in this study was created by measuring a plaster dental model with a Labo Scanner, which may result in some dimensional changes during impression taking and plaster hardening. Recently, intraoral scanners have gained popularity, and it is anticipated that future tooth movement analyses with PROI registration will achieve higher accuracy.

Limitations

Despite the promising findings, it is important to recognize the limitations of this study. The sample size was relatively small and specifically focused on the orthognathic surgery case population. This study excluded cases involving first premolar extraction or maximum anchorage with orthodontic implants, where significant lingual movement of the maxillary anterior teeth could lead to more pronounced changes in palatal morphology. Future studies with larger and more diverse samples may further validate the significance of these results.

## Conclusions

This study aimed to validate the efficacy of using DMs with reference to the palatal region of interest (PROI) for assessing orthodontic tooth movement (TM) compared to computed tomography (CT) models referenced to the cranial region of interest (CROI). Thirty-four patients with jaw deformities underwent DM and CT scans before and after presurgical orthognathic treatment. The 3D linear and angular measurements of TM were conducted using both DM and CT to assess reliability.

DM analysis with PROI registration exhibited high levels of reproducibility, with minimal standard errors in linear and angular measurements. CT analysis with CROI registration demonstrates similarly high reproducibility, with standard errors inferior to DM analysis. Bland-Altman analysis indicated agreement in linear and angular changes, with acceptable limits of agreement. DM offers distinct advantages including the absence of X-ray exposure, no metal artifacts, and the ability to generate high-resolution 3D models. The findings of the present study provide valuable insight into the use of 3D analyses in orthodontics and specifically highlight the validity of the use of PROI as a reference region for the evaluation of TM.
